# Caregiving for Dementia Patients during the Coronavirus Pandemic

**DOI:** 10.3390/jcm12072616

**Published:** 2023-03-30

**Authors:** Ana Claudia Damian, Adela Magdalena Ciobanu, Cristina Anghele, Ioana Raluca Papacocea, Mihnea Costin Manea, Floris Petru Iliuță, Constantin Alexandru Ciobanu, Șerban Papacocea

**Affiliations:** 1 Neuroscience Department, Discipline of Psychiatry, Faculty of Medicine, “Carol Davila” University of Medicine and Pharmacy, 020021 Bucharest, Romania; 2 Department of Psychiatry, “Prof. Dr. Alexandru Obregia” Clinical Hospital of Psychiatry, 041914 Bucharest, Romania; 3 Discipline of Physiology, Faculty of Medicine, “Carol Davila” University of Medicine and Pharmacy, 020021 Bucharest, Romania; 4 Department of Psychiatry and Psychology, “Carol Davila” University of Medicine and Pharmacy, 020021 Bucharest, Romania; 5 Faculty of Medicine, “Titu Maiorescu” University of Medicine and Pharmacy, 040441 Bucharest, Romania; 6 Department of Neurosurgery, Faculty of Medicine, “Carol Davila” University of Medicine and Pharmacy, 020021 Bucharest, Romania

**Keywords:** COVID-19, pandemic, corona, caregivers, dementia, life quality

## Abstract

The coronavirus pandemic has had a global impact on both mental and physical health. Caregiving has become more difficult during this time due to the quick spread of this respiratory disease, dread of the unknown, congested hospitals, and many restrictions, particularly for people with multiple comorbidities. We aimed to assess the impact of this pandemic on a group of caregivers of patients with dementia and their needs during this time. The study’s findings indicate that females assumed the role of the caregiver more often than men (88.5% of the participants) and scored lower on the life quality scale. The most often issue encountered during the pandemic was difficulty in accessing health care facilities (36%). Participants with a higher education level scored better in the physical (24.67, *p* = 0.01 and 24.48, *p* = 0.01) and mental health (20.67, *p* = 0.002; 19,82, *p* = 0.008) domains of the life quality test. The fear of COVID questionnaire showed a low level of concern in the category of participants with a high education level. Overall, this pandemic emphasizes the importance of social interaction and the possibilities to improve health care services through telemedicine. Caregivers could benefit from socialization and support programs as well as the early detection of affective disorders.

## 1. Introduction

Because the rapid spread of COVID-19 (Corona virus disease) in the community has outpaced the Chinese authorities’ ability to contain it, the World Health Organization declared a pandemic in March 2020 [1]. Due to its airborne transmission via secretions, this disease presented with symptoms such as fever, dry cough, and exhaustion, but it also developed more severe forms in the elderly and those with multiple comorbidities, generating acute respiratory syndrome, making it an international issue [2].

Official steps to prevent the spread of the virus generated concerns due to poor knowledge of the disease at the time, few available treatment options, fast spread of sickness with several cases of severe symptoms, and a lack of hospital beds. Nonetheless, the measures taken to contain the infectivity (such as wearing protective masks, frequent hand disinfection, maintaining the physical distance from others, declaring a state of national emergency with home confinement and limited exit between certain hours and only with a written statement) had the same effect [3].

One particularly vulnerable population is represented by the patients with major neurocognitive disorder (major NCD)/dementia, usually older than 65 years (characterized by memory loss, confusion, wondering, and difficulty in expressing thoughts that generate the need for assistance in everyday activities). These patients require full-time care, which is typically supplied by informal caregivers (carers that do not benefit from financial remuneration); without them, patients would have a lower quality of life and would be institutionalized. Patient’s spouses often fill this role, followed by sons/daughters, most often female, motivated by feelings such as love, reciprocity, and spiritual-religious fulfillment, a sense of duty, social pressure, or even greed [4,5,6].

Dementia is the fifth leading cause of death for older adults and there are over 50 million people globally suffering from this disease. In Romania, 300,000 people are diagnosed with Alzheimer’s disease (the most frequent form of dementia), with a low rate of detection (10–15%). Taking into consideration that for each patient there are 3–4 people implicated in caregiving, then the quality of life of at least one million Romanian residents is affected [7].

Costs of the dementia patient are direct (medical-diagnostic services, medication, emergency services, and non-medical-transportation, caregiving costs, additional expenses such as diapers, bed sheets) and indirect (the cost of the carer’s time while helping the patient) [8].

In Romania, the particularities of caregiving for dementia patients are as follows: (1) the low affordability of required medication for a healthy lifestyle—Vintila et al. reported that many Romanian elderlies invoked the lack of funds as their main reason for their absent healthy lifestyle; (2) Romania is one of the European countries allocating the smallest share of the gross domestic product to health care services; (3) awareness of dementia and its implications are low (suggested by the late management of the disease or the large number of comorbidities; (4) provision of services for dementia patients is still underdeveloped in Romania and lacks multidisciplinarity [9].

Caregivers of patients with major NCD had a difficult time during COVID because of the patient’s increased chance of acquiring a more severe form of the disease due to old age, multiple comorbidities, and a low capacity to adhere to the aforementioned guidelines and physical distance due to memory and attention deficiencies [10].

Furthermore, during the pandemic, the carers faced feelings of loneliness and social isolation, all of this contributing to chronic stress, feelings of weariness, an increased incidence of cardiovascular disease, lower immunity, and physical and financial issues [11,12,13,14,15].

Caregivers, often called “silent patients”, are the main focus of this article to better understand their needs due to their hardship throughout the pandemic. To our knowledge, this is the first paper of its kind and our goal was to analyze the effects of COVID on a group of informal caregivers of patients with dementia from Romania by evaluating their quality of life and perceived dread, and to determine the predictor factors of quality of life, and of the components of the quality of life.

## 2. Materials and Methods

For this descriptive, cross-sectional study in the Romanian population, we conducted a 6-month telephonic survey in 2022 with a series of questions addressed to a group of informal caregivers of patients with dementia admitted in the previous year at the “Prof. Dr. Al Obregia” Hospital in Bucharest, Romania.

Our research received ethical approval from the “Prof. Dr. Alexandru Obregia” Psychiatry Hospital Ethics Committee (approval number 73, 7 October 2021). In accordance with the Declaration of Helsinki and according to the country’s law, all participants included in the study provided written informed consent after the procedures of the study had been fully explained.

### 2.1. Participants

We selected a group of caregivers based on the following inclusion criteria:

- The participant is the main caregiver of the patient with major NCD—subtypes Alzheimer’s disease and vascular dementia, able to understand the language;
- Participants who provided consent and were able to understand the conditions necessary to participate in the study.

Exclusion criteria:

- Caregiver of patients with Lewy dementia;
- Caregiver who provides services for a fee;
- Caregiver already diagnosed with burnout;
- Caregivers currently diagnosed with an intellectual disability, psychiatric illness, or substance abuse in the last 12 months.

### 2.2. Procedure

The initial evaluation included a series of questions regarding the caregiver profile (age, gender, living environment, marital status, education, type of work activity), followed by a series of open questions regarding COVID (“Have you been diagnosed with COVID-19?”, “Have you ever been quarantined?”, “What were the problems encountered during the pandemic regarding the care of patients with major NCD?”). For the statistical process, the open answers regarding the problems encountered were coded as follows: 1 = No problems; 2 = Other problems (emotional, socio-professional and financial issues); 3 = Difficulty in accessing medical services; 4 = COVID infection; 5 = Death.

Subsequently, the following scales were applied:

- WHOQOL BREF (26 items—consisting of four areas: physical health (seven questions related to mobility, daily activities, functional capacity, energy, pain and sleep), mental health (six questions related to self-image, negative thoughts, positive attitudes, mentality, ability to learn, focus, religion and mental status), social relationships (three questions about personal relationships, social support, sex life), and environmental health (eight questions about financial resources, safety, health and social services, the environment in which they live, opportunities to acquire new knowledge and skills, recreation, the environment and means of transport), with scoring from 1–5. This widely used scale was developed by the World Health Organization for cross-cultural comparison. The reliability was assessed using the Cronbach’s alpha coefficient (0.91) and according to the convergent validity results, the correlation coefficient values were strongly associated at 0.01 [16,17].

The 4 domains were coded as follows: Domain 1 = Physical health; Domain 2 = Mental health; Domain 3 = Social relations; Domain 4 = Environment.

- Fear of COVID-19 scale (7 items—response options from 1–5 (strongly disagree–completely agree) was developed in Iran by a group a researchers for measuring anxiety and fear of COVID using seven questions with five possible answers. As a result, the total score ranged between 7 and 35. With robust psychometric properties, this scale has been shown to be valid and reliable in assessing the fear of COVID in the general population (internal consistency α = 0.82, test–retest reliability ICC = 0.72) [18,19].

After collecting the information, the data were analyzed in SPSS version 20. For the quantitative data, the mean and the standard deviation were used as well as the Pearson correlations, and for the qualitative data, the frequency and the percentage were used. Moreover, for the interpretation of the results, several *t*-tests and ANOVA (analysis of variance) were selected, and linear regressions were employed to analyze the predictor variables of each dependent variable, building regression models.

## 3. Results

We contacted a number of 240 caregivers to address the invitation to participate in this study, out of which 42 were not eligible to participate because they were already diagnosed with a psychiatric condition or provided services for a fee. The remaining 198 caregivers were included in the study. However, 18 of them withdrew their consent along the way (four of them could not be reached by phone and 14 of them had connection difficulties), and 24 of them lost their caregiver status (they were no longer a caregiver because the patient died or they went to several dementia care homes).

### 3.1. Description of the Group

A total of 156 participants responded to our questionnaire, most of them women (88.5%, N = 138), living in Bucharest (41%, N = 64), with a high level of education (university 53.8%, N = 84), married (57.6%, N = 90) or in a relationship (20.5, N = 32), and with a job that required a physical presence (41%, N = 64).

### 3.2. Questions Regarding COVID

Most of the participants had not been quarantined (63%, N = 98) or diagnosed with the disease (68%, N = 106).

During COVID, most of the participants reported difficulty in accessing the medical services (35.9%, N = 56) and a concerning amount reported the death of the patient they were caring for (19.3%, N = 30).

When enquired about other difficulties, socio-professional issues were reported by the majority of the participants (64%, N = 100), followed by emotional issues (34.6%, N = 54).

### 3.3. Questionnaires

Based on the *t*-test, there was a significant difference between gender and the social relations dimension of the WHOQOL26 (*t* = 2.03, *p* = 0.04), specifically, the male gender scored higher (11.22 vs. 9.77) (Table 1).

Based on the mean values calculated for the four WHOQOL domains, the participants scored lowest on the social relations domain (9.94), followed by mental health (20.76). The mean score for the fear of COVID questionnaire was 17.3 (Table 2).

The ANOVA test revealed a significant difference regarding the education level and total scores for physical health (F(2.152) = 4.64, *p* = 0.01), mental health (F(2.153) = 6.18, *p* = 0.003), and quality of life (F(2.153) = 4.61, *p* = 0.01). University and post university graduates scored higher in the physical health domain (24.67, *p* = 0.01 and 24.48, *p* = 0.01) and mental health domain (20.67, *p* = 0.002; 19.82, *p* = 0.008) compared to high school graduates (Table 3).

**Figure 1:**
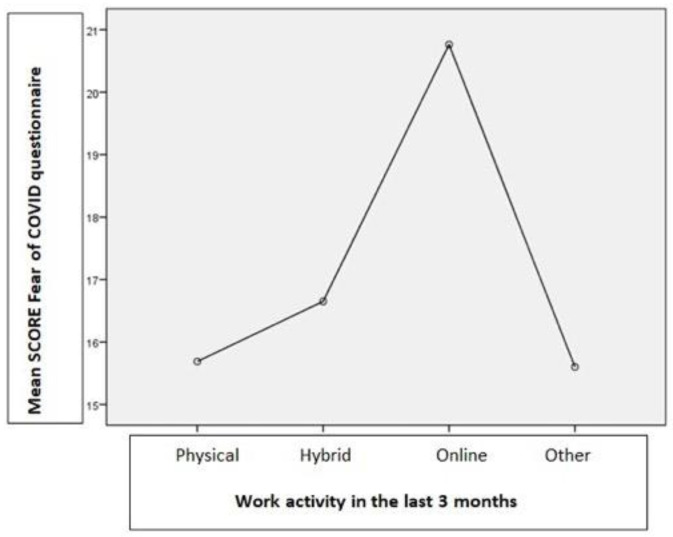
Mean score of the participants to the Fear of COVID questionnaire based on their work activity.

Fear of COVID has been associated with work type activity. According to the ANOVA test using the total score of the Fear of COVID questionnaire (F(3.152) = 6.96, *p* = 0.001), participants who required a physical presence to work scored lower in the test compared to the ones who worked online (20.76, *p* = 0.001), or even hybrid (16.65, *p* = 0.01). A detailed chart of the mean scores can be found in Figure 1.

The caregivers who reported emotional problems during this period scored lower in the physical health (23.85, *p* = 0.03), mental health (19.59, *p* = 0.04), social relations (8.63, *p* = 0.001), and quality of life (78.96, *p* = 0.01) in general, but scored higher in the Fear of COVID scale (16.65, *p* = 0.01) (Table 4).

Caregivers reporting socio-professional issues scored lower in the socio-professional dimension (9.52, *p* = 0.01) and quality of life (81.40, *p* = 0.03), but scored higher on the Fear of COVID scale (18.64, *p* = 0.001) (Table 5).

**Figure 2:**
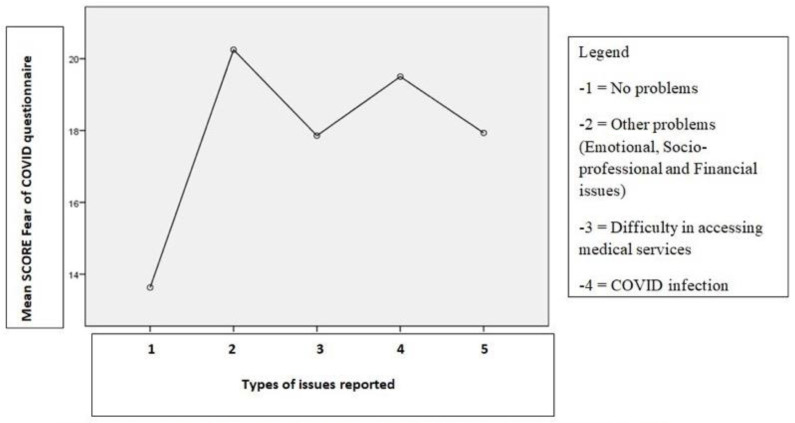
Mean score for the Fear of COVID questionnaire based on the issues reported by the participants.

Caregivers reporting financial problems scored lower in the Fear of COVID scale (14.33, *p* = 0.03) and social relations dimension (7.67, *p* = 0.04) (Table 6). Overall, the category of participants with emotional, socio-professional, and financial issues (category 2) as well as the participants with COVID infection (category 4) recorded the highest scores on the Fear of COVID questionnaire (Figure 2).

In order to determine the predictor factors of quality of life, and of the components of the quality of life, several linear regressions were employed to analyze the predictor variables of each dependent variable, building regression models (Appendix A).

For quality of life, all of the components had positive and strong relationships because they were components of the quality of life, and for COVID, there were weak negative relationships, highlighting the fact that fear of COVID and the health of the environment has no relationship, so they do not influence one another (Table 7).

Quality of life (F(2.153) = 8.75, *p* = 0.001) was influenced by the fear of COVID and the emotional problems of the caregivers. Thus, both fear of COVID (beta = −0.25, *t* = −3.29, *p* = 0.001) and the emotional problems (beta = −0.16, *t* = −2.06, *p* = 0.04) of caregivers negatively influenced the quality of life. Moreover, the variance explained by the fear of COVID and the emotional problems was 10% (Table 8).

Physical health (beta = −0.36, *t* = −4.83, *p* = 0.001) and mental health (beta = −0.31, *t* = −4.05, *p* = 0.001) were negatively influenced by the fear of COVID, and social relationships were negatively influenced by emotional problems (beta = −0.33, *t* = −4.49, *p* = 0.001) and socio-professional problems (beta =−0.20, *t* = −2.67, *p* = 0.008) of the participants. Moreover, the explained variance of physical health by the fear of COVID of caregivers was 13%, and the fear of COVID explained 9% of the variance of psychological health. Emotional and socio-professional problems explained 15% of the health variance of social relations.

## 4. Discussion

Overall, the majority of respondents to our questionnaire defined the typical caregiver as a female, 40–45 years old, married, with a high education, living in an urban location, and having a profession that needs a physical or hybrid (physical and online) presence. These results are consistent with the existing literature where women usually assumed the role of caregiving [20].

Even though the male participants were poorly represented (only 11%), they scored higher on the WHOQOL-26, suggesting a better quality of life than women. Cohen Steven A. et. al. debated in their paper whether the burden of caregivers differed by gender. Females, for example, are more likely to take on the role of caretaker, even when the duty is emotionally or physically difficult, in comparison to men. This could explain why the impact on quality of life is higher in the former category [21].

Our results suggest that most of the participants had not been diagnosed with this disease or quarantined, scoring low on the Fear of COVID scale. One possible explanation could be their high level of education, which provided them a better understanding of the situation and the importance of restrictions. The ANOVA test suggested that participants with a higher education level scored better in the physical and mental health domains. Another explanation could be that the fear of not spreading the disease to the already vulnerable patient made them even more careful.

During this difficult time, the participants reported that they had difficulties in accessing medical services, which is not surprising considering that this pandemic has led to the breakdown of health care systems worldwide and a decrease in the quality of health care due to the overwhelmed wards or intensive care units. Amongst the most common problems described are discontinuation of specialized medical care, difficulty accessing hospitals, or even appointments and frequent COVID antigen testing. Similarly, a recent European study described some of the frequent issues encountered: some hospitals had to reschedule non-urgent visits for safety measures, or patients cancelled because of the fear of infection [22,23]. Werner et. al. concluded in their study that more than half of the participants (approximately 35 families of caregivers in Israel) were forced to postpone the use of medical services (for patients or for themselves and their families), which was mainly described among families with a lower financial income [24].

Additional problems include the stress felt as a result of the patient’s behavioral changes and worsening of cognition, the appearance of negative feelings, enforcing COVID restrictions for a hardly cooperative patient, and the lack of support [25,26].

Penteado et al. studied 71 patients with dementia and discovered that the most prevalent neurocognitive symptoms that developed or worsened during the pandemic were mood disorders, apathy, and sleep disorders. This deterioration can be explained by social isolation, feelings of loneliness, sudden changes in routine and leisure activities, lack of stimulating activities, and a lack of sufficient therapeutic follow-up. As a result, caregivers felt compelled to seek institutionalization and reported reduced tolerance to frustration and increased levels of stress, all of which had a detrimental influence on their life [27,28,29,30,31,32,33,34,35].

Another concern mentioned by caregivers in our study was the death of the patient they were caring for, some of whom has previously required hospitalization and were COVID positive (during this challenging time, family visits were prohibited).

A few participants reported the occurrence of other problems such as fear of not spreading the virus to loved ones, stress, irritability and feelings of helplessness; socio-professional problems at work due to the fact that many employers had chosen an online work environment, but the patients required permanent supervision; or even staff restructuring; the difficulty of helping patients understand and follow the rules imposed during the pandemic; the strict restrictions that gave the feeling of lack of freedom, fear of unknown, lack of socialization and slow vaccination process.

In the existing literature, the fear of transmitting the disease to the patient or the continuous effort to persuade them to follow the new hygiene and physical restraint measures has been shown to increase the level of anxiety or even affective disorders such as depression because of the patient’s cognitive impairment [21,27,36,37,38,39,40,41,42].

Another issue reported by the caregivers is maintaining their socio-professional status, which is understandable given that the majority of the respondents had jobs to attend to, social isolation measures, and multiple restrictions, giving the impression of loneliness. This is consistent with their test results in the WHOQOL-26, since the lowest scores were in the social relations domain. The same participants reported an increasing fear of COVID.

Despite the fact that the mean score for the Fear of COVID scale indicated a low level of concern for the disease, caregivers who reported emotional problems scored higher on this test, suggesting an important impact to their lives, which is consistent with their low scores on the quality of life questionnaire.

However, the predictor factors in all cases explained very little of the variance in the dependent variables, therefore, they are weak factors to evaluate the quality of life during the pandemic, implying that additional predictor factors for these members should be utilized.

Another study conducted in Spain with 106 families of caregivers during the pandemic described the negative evolution of caregivers, which was characterized by anxiety, mood disorders, sleep and eating disorders. No less than 46% of them described that recreational activities were the ones that they would have needed the most help with, but also basic tasks such as dressing, eating, and maintaining personal hygiene. In addition, in this study, caregivers described the methods adopted to cope with this situation such as increasing medication, going out on the balcony, looking at images of loved ones, dancing, listening to music, family video conferencing, and recreational games. When asked about various alternatives that they wished they had received to reduce their stress levels, they suggested official caregivers or the support of family and friends [43].

Our findings suggest the need to implement specific programs for caregivers to learn “coping” skills, socialization, support groups, online interventions to reduce stress, anxiety and depression [37,44,45,46]. A rigorous examination is also required to detect caregivers with symptoms of anxiety, depression, or burnout, particularly in females and caregivers co-living with the patient where apparently the risk is higher [27,47,48,49,50,51,52,53,54].

Additionally, telemedicine through video conferencing should be further explored, especially in times when social interaction is limited. Telemedicine would enable video/telephonic appointments between health care practitioners and their patients, thus reducing physical contact, reduce travel time, costs, and time off work. It could be used for home monitoring, videophone interpretation while on a consult, online educational programs, and telephonic triage before admission, even robotic surgery. The requirements for telehealth are good Internet access and broadband mobile technology (at least 4G), with various forms of delivery (live video, store-and-forward, mobile health, or remote patient monitoring). However, there is limited evidence on these benefits, which requires more research [55,56].

Examples of using telemedicine to help caregivers include online educational programs for caregivers, telephonic triage of the caregivers for psychiatric evaluation, online support groups, and video appointments for the patients.

Several limitations of this study include: (1) the small sample of participants being insufficient for statistical generalization; (2) telephonic surveys, even though widely used and advantageous compared to face-to-face interviewing, lack representativeness; another issue could be the difficulty in maintaining a connection or the limitation of question complexity addressed over the phone; (3) reliability of the survey data as participants may not feel motivated to deliver accurate answers, or uncomfortable in offering responses that could portray them negatively.

## 5. Conclusions

The study’s findings indicate the usual caregiver profile: female, 40–45 years old, married, with a high education, living in an urban setting, and having a job that requires a physical or hybrid presence.

The majority of the participants reported difficulty in accessing health care facilities. Participants with a higher education level scored better in the physical and mental health domains. The Fear of COVID questionnaire showed a medium level of concern.

Fear of COVID negatively influenced physical and mental health and emotional problems lowered social interaction. However, fear of COVID influenced the quality of life and the appearance of emotional issues.

After reviewing the literature and assessing the negative evolution of patients with major NCDs (behavioral disorders, cognitive impairment) during social isolation, we can conclude that socialization and support programs for caregivers and patients as well as the early detection of affective disorders or burnout in caregivers are required. Comparative research on the evolution of quality of life across time is needed.

Telemedicine could facilitate caregivers by reducing travel time, costs, and time off work, the implementation of group support, educational programs, and the triage of psychiatric disorders in caregivers. Taking a step back, one positive aspect of this pandemic has to be the explosive growth of telemedicine, which we can only hope will improve health care.

## Figures and Tables

**Table 1:** Mean scores of the questionnaires.

	Age	Total Score Fear of COVID	Total Score Physical Health	Total Score Mental Health	Total Score Social Relations	Total Score Environment	Total Score WHOQOL26
Mean	43.62	17.29	24.99	20.76	9.94	27.81	83.49
Std. Deviation	10.236	6.180	5.295	5.255	2.875	5.616	16.709

**Table 2:** Correlations between the WHOQOL26 mean scores and gender.

Score	Gender	N	Mean	Std. Deviation
Physical health total score	Male	18	27	4.029304
	Female	138	24.72464	5.395007
Mental health total score	Male	18	22.88889	4.86148
	Female	138	20.47826	5.257206
Social relations total score	Male	18	11.22222	2.51011
	Female	138	9.768116	2.885377
Environment total score	Male	18	28.44444	3.433781
	Female	138	27.72464	5.844416
Quality of life total score	Male	18	89.55556	12.65944
	Female	138	82.69565	17.04464

**Table 3:** Cross-reference between educational level and the participants’ responses to the questionnaires.

		Sum of Squares	df	Mean Square	F	Sig.
Total score—Fear of COVID	Between Groups	71.843	2	35.922	0.940	0.393
	Within Groups	5848.593	153	38.226		
	Total	5920.436	155			
Total score—Physical health	Between Groups	248.825	2	124.412	4.646	0.011
	Within Groups	4097.149	153	26.779		
	Total	4345.974	155			
Total score—Mental health	Between Groups	320.087	2	160.043	6.182	0.003
	Within Groups	3960.657	153	25.887		
	Total	4280.744	155			
Total score—Social relations	Between Groups	41.180	2	20.590	2.540	0.082
	Within Groups	1240.179	153	8.106		
	Total	1281.359	155			
Total score—Environment	Between Groups	120.585	2	60.293	1.935	0.148
	Within Groups	4767.645	153	31.161		
	Total	4888.231	155			
Total score—WHOQOL-26	Between Groups	2461.633	2	1230.816	4.614	0.011
	Within Groups	40,813.342	153	266.754		
	Total	43,274.974	155			

**Table 4:** Cross-reference between the total scores of participants reporting emotional issues.

Score	Emotional Issues	N	Mean	Std. Deviation
Total score—Fear of COVID	Yes	54	18.56	4.932
	No	102	16.63	6.675
Total score—Physical health	Yes	54	23.85	4.486
	No	102	25.59	5.605
Total score—Mental health	Yes	54	19.59	4.847
	No	102	21.37	5.380
Total score—Social relations	Yes	54	8.63	2.475
	No	102	10.63	2.842
Total score—Environment	Yes	54	26.89	5.765
	No	102	28.29	5.502
Total score—WHOQOL26	Yes	54	78.96	15.138
	No	102	85.88	17.073

**Table 5:** Cross-reference between the total scores of participants reporting socio-professional issues.

Score	Socio-Professional Issues?	N	Mean	Std. Deviation
Total score—Fear of COVID	Yes	100	18.64	6.684
	No	56	14.89	4.250
Total score—Physical health	Yes	100	24.42	5.078
	No	56	26.00	5.566
Total score—Mental health	Yes	100	20.12	4.785
	No	56	21.89	5.880
Total score—Social relations	Yes	100	9.52	2.668
	No	56	10.68	3.099
Total score—Environment	Yes	100	27.34	5.400
	No	56	28.64	5.940
Total score—WHOQOL26	Yes	100	81.40	15.366
	No	56	87.21	18.432

**Table 6:** Cross-reference between the total scores of participants reporting financial issues.

Score	Financial Issues?	N	Mean	Std. Deviation
Total score—Fear of COVID	Yes	6	14.33	2.582
	No	150	17.41	6.256
Total score—Physical health	Yes	6	26.33	2.251
	No	150	24.93	5.378
Total score—Mental health	Yes	6	21.67	3.615
	No	150	20.72	5.316
Total score—Social relations	Yes	6	7.67	2.251
	No	150	10.03	2.866
Total score—Environment	Yes	6	31.00	5.865
	No	150	27.68	5.588
Total score—WHOQOL26	Yes	6	86.67	12.691
	No	150	83.36	16.870

**Table 7:** The Pearson coefficients of the dependent variables.

Variables	1	2	3	4	5	6
1. Quality of life	1	0.91 **	0.90 **	0.85 **	0.83 **	−0.27 **
2. Physical health	0.91 **	1	0.84 **	0.72 **	0.62 **	−0.36 **
3. Psychological health	0.90 **	0.82 **	1	0.76 **	0.59 **	−0.31 **
4. Social relations health	0.85 **	0.72 **	0.76 **	1	0.64 **	−0.16 *
5. Environment health	0.83 **	0.62 **	0.59 **	0.64 **	1	NS
6. Fear of COVID	−0.27 **	−0.36 **	−0.31 **	−0.16 *		1

** *p* < 0.001; * *p* < 0.05; NS—not statistically significant.

**Table 8:** The predictors of the regression models.

No. of Model	Dependent Variable	Independent Variables	R^2^	Beta	*t*	*p*
1.	Quality of life	Fear of COVID	0.10	−0.25	−3.29	0.001
		Emotional problems of the family		−0.16	−2.06	0.04
2.	Physical health component	Fear of COVID	0.132	−0.363	−4.83	0.001
3.	Psychological health component	Fear of COVID	0.09	−0.31	−4.05	0.001
4.	Social relations health component	Emotional problems of the family	0.15	−0.33	−4.49	0.001
		Socio-professional problems of the family		−0.20	−2.67	0.008

**Figure A1:**
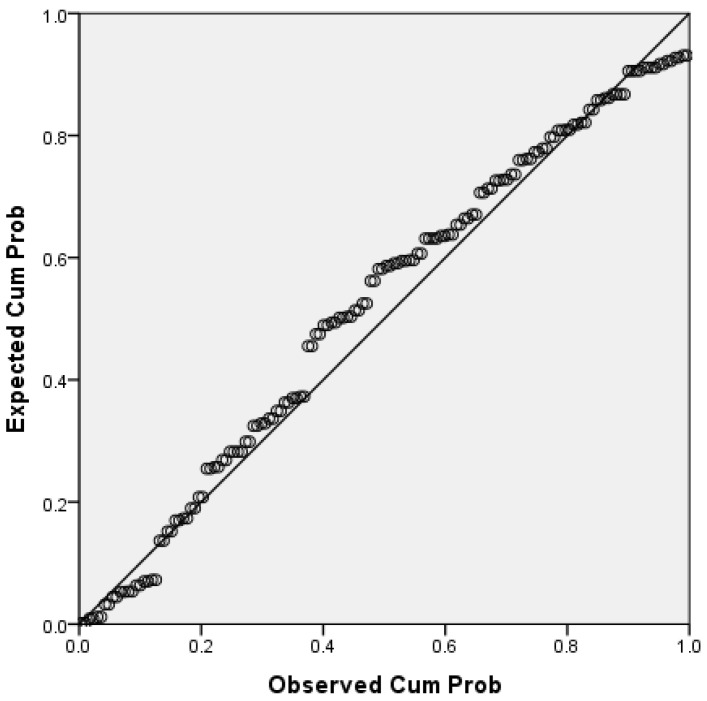
Normal Q–Q plot for the quality of life model residuals.

**Figure A2:**
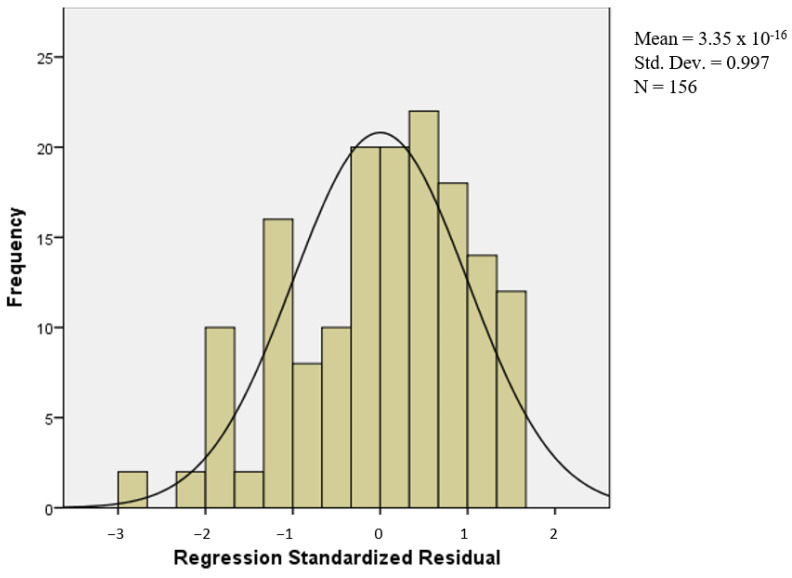
Histogram using physical health as the dependent variable.

**Figure A3:**
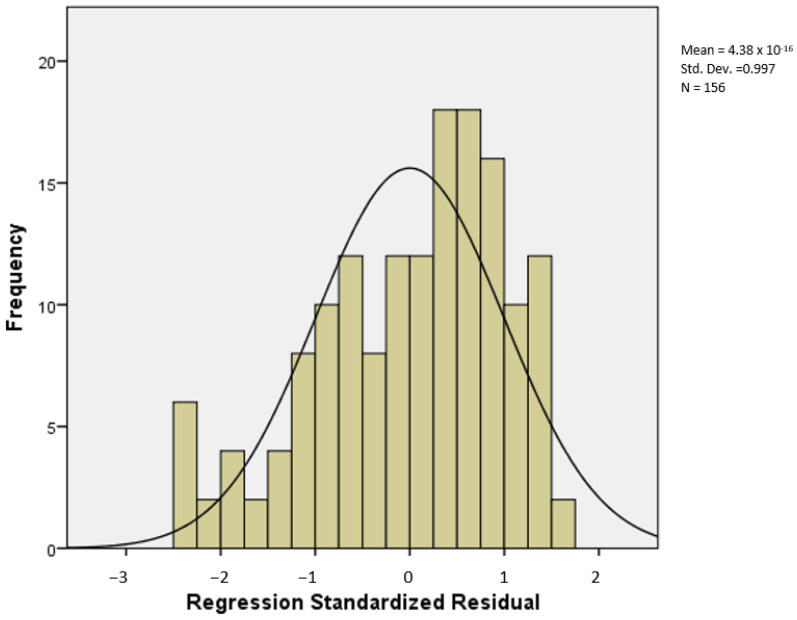
Histogram using mental health as the dependent variable.

**Figure A4:**
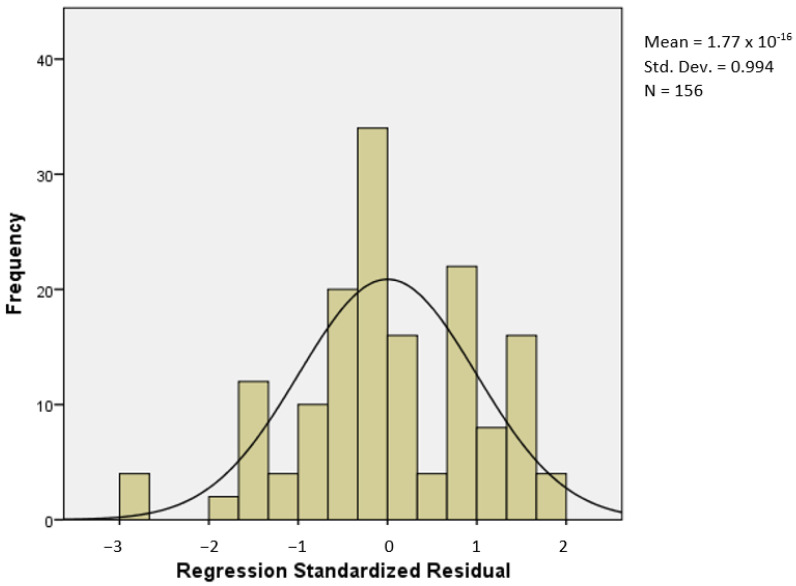
Histogram using social relationships as the dependent variable.

## Data Availability

All data reported in the article are available in anonymized form upon request.
